# Biological age is superior to chronological age in predicting hospital mortality of the critically ill

**DOI:** 10.1007/s11739-023-03397-3

**Published:** 2023-08-28

**Authors:** Kwok M. Ho, David J. Morgan, Mason Johnstone, Cyrus Edibam

**Affiliations:** 1 Department of Intensive Care Medicine, Fiona Stanley Hospital, Perth, WA Robin Warren Drive, 6150 Australia; 2 University of Western Australia, Perth, WA 6009 Australia; 3 Murdoch University, Perth, WA 6150 Australia

**Keywords:** Chronological age, Comorbidity, Phenotypical age, Prognosis, Outcome, Severity of illness

## Abstract

Biological age is increasingly recognized as being more accurate than chronological age in determining chronic health outcomes. This study assessed whether biological age, assessed on intensive care unit (ICU) admission, can predict hospital mortality. This retrospective cohort study, conducted in a tertiary multidisciplinary ICU in Western Australia, used the Levine PhenoAge model to estimate each patient’s biological age (also called PhenoAge). Each patient’s PhenoAge was calibrated to generate a regression residual which was equivalent to biological age unexplained by chronological age in the local context. PhenoAgeAccel was a dichotomized measure of the residuals, and its presence suggested that one was biologically older than the corresponding chronological age. Of the 2950 critically ill adult patients analyzed, 291 died (9.9%) before hospital discharge. Both PhenoAge and its residuals (after regressing on chronological age) had a significantly better ability to differentiate between hospital survivors and non-survivors than chronological age (area under the receiver-operating-characteristic curve 0.648 and 0.654 vs. 0.547 respectively). Being phenotypically older than one’s chronological age was associated with an increased risk of mortality (PhenoAgeAccel hazard ratio [HR] 1.997, 95% confidence interval [CI] 1.568–2.542; p = 0.001) in a dose-related fashion and did not reach a plateau until at least a 20-year gap. This adverse association remained significant (adjusted HR 1.386, 95% CI 1.077–1.784; p = 0.011) after adjusted for severity of acute illness and comorbidities. PhenoAgeAccel was more prevalent among those with pre-existing chronic cardiovascular disease, end-stage renal failure, cirrhosis, immune disease, diabetes mellitus, or those treated with immunosuppressive therapy. Being phenotypically older than one’s chronological age was more common among those with comorbidities, and this was associated with an increased risk of mortality in a dose-related fashion in the critically ill that was not fully explained by comorbidities and severity of acute illness.

**Supplementary Information:**

The online version contains supplementary material available at 10.1007/s11739-023-03397-3.

## Introduction

Aging population is a major public health problem in many developed countries. How to improve quality of health and life of the aging population is an emerging research paradigm [1]. Chronological age has an important association with the biological functions of all mammals but longevity of each species as well as between different individual organisms within the same species is highly variable [2]. Unlike a car or a machine, different living systems have acquired some in-built strategies to modulate and adapt to cellular breakdowns and mutations, even though these strategies will eventually fail. Different biological and pathogenic mechanisms have been proposed to explain why aging or senescence occurs, and whether it is preventable. Deteriorations in mitochondrial functions or activities, DNA methylation, and spontaneous somatic mutations – including Clonal Hematopoiesis of Indeterminate Potential (CHIP) – are currently believed to be most probable key pathogenic mechanisms [3].

Regardless of the precise mechanisms that lead to cellular and organ senescence, a certain phenotypical manifestation of aging can be observed which appears to be more predictive of a person’s health and even longevity than chronological age. Frailty is a well-recognized phenotypical feature of aging [4]. Recently, advanced computing capability including the use of artificial intelligence has allowed researchers to identify biomarkers of DNA methylation and quantify biological age of individuals. Evidence suggests that phenotypical or biological age of a person is a dynamic ‘epigenetic clock’ – which can be dialled forward with diseases and health events such as surgery, pregnancy, or COVID-19 infection [5], and conversely regressed backward with interventions [6] – as compared to chronological age. A phenotypical or biological age model – the Levine PhenoAge — based on data input from nine commonly used blood tests was published in 2018 and it was subsequently validated to be predictive of 10-year survival, cognitive dysfunction, and diabetes mellitus over and beyond a person’s chronological age, education background, socioeconomic status and smoking habit, including among those who are older than 80 years-old [7–10]. Furthermore, somatic mutations, such as CHIP, are strongly linked to age acceleration in multiple biological age clocks, including the Levine PhenoAge model [3], suggesting that epigenetic aging may be used to identify a population at high risk for adverse health outcomes and who may be a target for clinical interventions.

If phenotypical or biological age is proven to be better than chronological age in reflecting the health status of a person, it would make sense to use biological age instead of chronological age for risk adjustment in health outcome studies. Currently, the ability of biological age to predict outcomes of critically ill patients has not been assessed and its utility remains uncertain. We hypothesized that biological or phenotypical age was more important than chronological age in determining mortality of the critically ill and being biologically older than one’s chronological age would be associated with worse mortality outcome. In this study, we aimed to compare the ability of biological age to chronological age in predicting hospital mortality of critically ill patients. Specifically, we also aimed to assess whether being phenotypically older than one’s chronological age was more common among those with pre-existing chronic health conditions, and phenotypical age more than chronological age could predict mortality after adjusted for severity of acute illness and comorbidities.

## Materials and methods

After obtaining research approvals from the Hospital Quality Improvement Unit and Clinical Information System administrative committee (approval number 49386 on February 28, 2023: Performance of the current risk adjustment models with and without PhenoAge in intensive care patients), de-identified data of critically ill patients admitted to the intensive care unit (ICU) of Fiona Stanley Hospital in Western Australia between June 1, 2015 and June 30, 2021 were retrieved from the Clinical Information System. Procedures were followed in accordance with the ethical and data protection standards of the approving committees and with the Helsinki Declaration of 1975. Fiona Stanley Hospital is a tertiary teaching hospital, and its 40-bed ICU admits critically ill patients of most medical and surgical specialties including heart and lung transplantation and burns.

In this retrospective cohort study, we used the Levine’s PhenoAge model to estimate the phenotypical or biological age of each patient [9, 10]. The Levine PhenoAge model was derived using prospectively collected clinical data of the third National Health and Nutrition Examination Survey (NHANES) III [9] and uses biomarkers that reflect DNA methylation which is believed to be one of the key pathogenic processes of aging. It utilizes chronological age as well as nine blood tests are needed to calculate the PhenoAge of the patients. The accuracy of this model was validated using the NHANES IV data (n = 14,008) [10]. The nine blood tests used in the Levine PhenoAge model include C-reactive protein, glucose concentration, mean red blood cell volume, red blood cell distribution width, albumin concentration, creatinine concentration, lymphocyte percentage, alkaline phosphatase, and white cell count. The equations needed to compute PhenoAge are described in Online supplement Table 1.

**Table 1:** Characteristics of the cohort (n = 2950) and differences between hospital survivors and non-survivors

Variable	All patients (N = 2950)	Survivors (n = 2659, 90.1%)	Non-survivors (n = 291, 9.9%)	p value^#^
Chronological age, years (IQR)	63 (49–72)	62 (48–72)	65 (53–74)	0.008
Male, no. (%)	1703 (57.7)	1530 (57.5)	173 (59.5)	0.782
Body mass index, kg/m^2^ (IQR)	28 (24–33)	28 (24–33)	28 (24–33)	0.998
PhenoAge, years (IQR)	91.5 (74.5–109.0)	90.3 (73.6–107.0)	104.6 (87.2–121.0)	0.001
Difference between phenoage and chronological age, years (IQR)	27.5 (17.8–40.7)	26.5 (17.3–39.6)	36.2 (25.7–48.4)	0.001
PhenoAgeAccel*, no. (%)	1275 (43.2)	1087 (40.9)	188 (64.6)	0.001
Elective surgical admission, no. (%)	808 (27.4)	785 (29.5)	23 (7.9)	0.001
Chronic respiratory disease, no. (%)^a^	120 (4.1)	98 (3.7)	22 (7.6)	0.001
Chronic cardiovascular disease, no. (%)^a^	228 (7.7)	193 (7.3)	35 (12.0)	0.004
End-stage renal failure, no. (%)^a^	126 (4.3)	112 (4.2)	14 (4.8)	0.631
Cirrhosis, no. (%)^a^	64 (2.2)	50 (1.9)	14 (4.8)	0.001
Immune disease, no. (%)^a^	66 (2.2)	58 (2.2)	8 (2.7)	0.534
Immunosuppressed, no. (%)^a^	237 (8.0)	197 (7.4)	40 (13.8)	0.001
Lymphoma, no. (%)^a^	57 (1.9)	45 (1.7)	12 (4.1)	0.004
Leukemia, no. (%)^a^	53 (1.8)	45 (1.7)	8 (2.7)	0.198
Metastatic cancer, no. (%)^a^	162 (5.5)	152 (5.7)	10 (3.4)	0.105
AIDS, no. (%)^a^	5 (0.2)	3 (0.1)	2 (0.7)	0.024
Diabetes mellitus, no. (%)**	247 (23.2)	226 (23.9)	21 (18.1)	0.166
Admission diagnosis, no. (%)				0.001
Cardiac surgery	183 (6.2)	175 (6.6)	8 (2.7)	
Burns	20 (0.7)	17 (0.6)	3 (1.0)	
Liver or pancreatic surgery	262 (8.9)	257 (9.7)	4 (1.7)	
Upper gastrointestinal tract surgery	139 (4.7)	133 (5.0)	6 (2.1)	
Ascular surgery	80 (2.7)	77 (2.9)	3 (1.0)	
Urological surgery	27 (0.9)	27 (1.0)	1 (0)	
Pneumonia/aspiration	298 (10.1)	259 (9.7)	39 (13.4)	
Sepsis	726 (24.6)	647 (24.3)	79 27.1)	
Cardiac arrest	94 (3.2)	64 (2.4)	30 (10.3)	
Bowel obstruction/perforation	136 (4.6)	127 (4.8)	9 (3.1)	
Drug overdoses/self-harm	62 (2.1)	61 (2.3)	2 (0.3)	
Stroke/coma/seizures	148 (5.0)	131 (4.9)	17 5.8)	
Obstructive airway or interstitial lung disease	102 (3.5)	91 (3.4)	10 3.8)	
Acute kidney injury	65 (2.2)	57 (2.1)	8 (2.7)	
Cardiogenic shock/heart failure	122 (4.1)	99 (3.7)	23 7.9)	
Others	486 (16.4)	437 (16.4)	49 (16.8)	
APACHE II score (IQR)	15 (11–21)	15 (11–20)	25 (19–31)	0.001
APACHE II score > 15, no. (%)	1459 (49.5)	1208 (45.4)	251 (86.3)	0.001
Invasive ventilation within 24 h of admission, no. (%)	1302 (44.1)	1102 (41.1)	200 (68.7)	0.001
Requiring vasopressor within 24 h of admission, no. (%)	1464 (49.6)	1231 (46.3)	233 (80.0)	0.001
Requiring renal replacement therapy within 24 h of admission, no. (%)	254 (8.6)	212 (8.0)	79 (27.2)	0.001
ICU stay, days (IQR)	3 (1–5)	3 (1–5)	4 (2–8)	0.001
Hospital stay, days (IQR)	10 (6–18)	10 (7–19)	6 (2–15)	0.001

*PhenoAgeAccel is a dichotomized estimate to reflect whether the biological age of the patient is older than the chronological age while accounting for the PhenoAge of the whole cohort by regressing PhenoAge on chronological age; a positive residual means a patient’s phenotypical or biological age is older than their chronological age compared to other patients included in this study. ^a^All these comorbidities are defined according to the Acute Physiology and Chronic Health Evaluation (APACHE) II model, immunosuppressed is defined as receiving high dose systemic steroids or chemotherapy within thirty days of ICU admission. IQR, interquartile range. ICU, intensive care unit. **Only 1063 patients had documented diabetes mellitus data in the clinical information system. All continuous data are in median with IQR. ^#^P values were derived from either Chi Square or Mann-Whitney test

In addition to calculating the PhenoAge, we also assessed the predictive ability of three PhenoAge-related age measures, including (a) the absolute difference between PhenoAge and chronological age, (b) the residuals from regressing PhenoAge on chronological age using the whole cohort of patients and (c) PhenoAgeAccel – when the residual is positive [9]. The scientific rationale for assessing the latter two PhenoAge related measures are as follows. By including chronological age as a predictor, PhenoAge tends to diverge positively from chronological age as the latter is increasing; using residuals from regressing PhenoAge on chronological age would avoid this potential problem. Another distinct advantage of using the residuals from the regression instead of the absolute difference between PhenoAge and chronological age is that the residuals are indexed to other patients within the same context or study, like a concept called re-calibration based on local context. Most, if not all, existing ICU prognostic models have not incorporated this feature in a standard fashion.

PhenoAgeAccel is a dichotomized representation of the residuals of the regression, patients who have a positive residual signify their phenotypical or biological age years are older than their corresponding chronological age in comparison to all patients included in the study. The distribution of the residuals of the regression relationship between chronological age and PhenoAge of the current study patients, and a scatter plot showing how chronological age was related to PhenoAge for each patient are described in Online supplement Fig. 1a and b, respectively. The constant of the regression line represents the recalibrated constant of the relationship between PhenoAge and the chronological age in the current study context. As such, PhenoAgeAccel after indexed to the rest of the patients in this study was considered to exist only for those who had a PhenoAge in excess of 33.4 years (which is the Y-intercept of the regression line) (Online supplement Fig. 1b) above their corresponding chronological age.

**Fig. 1:**
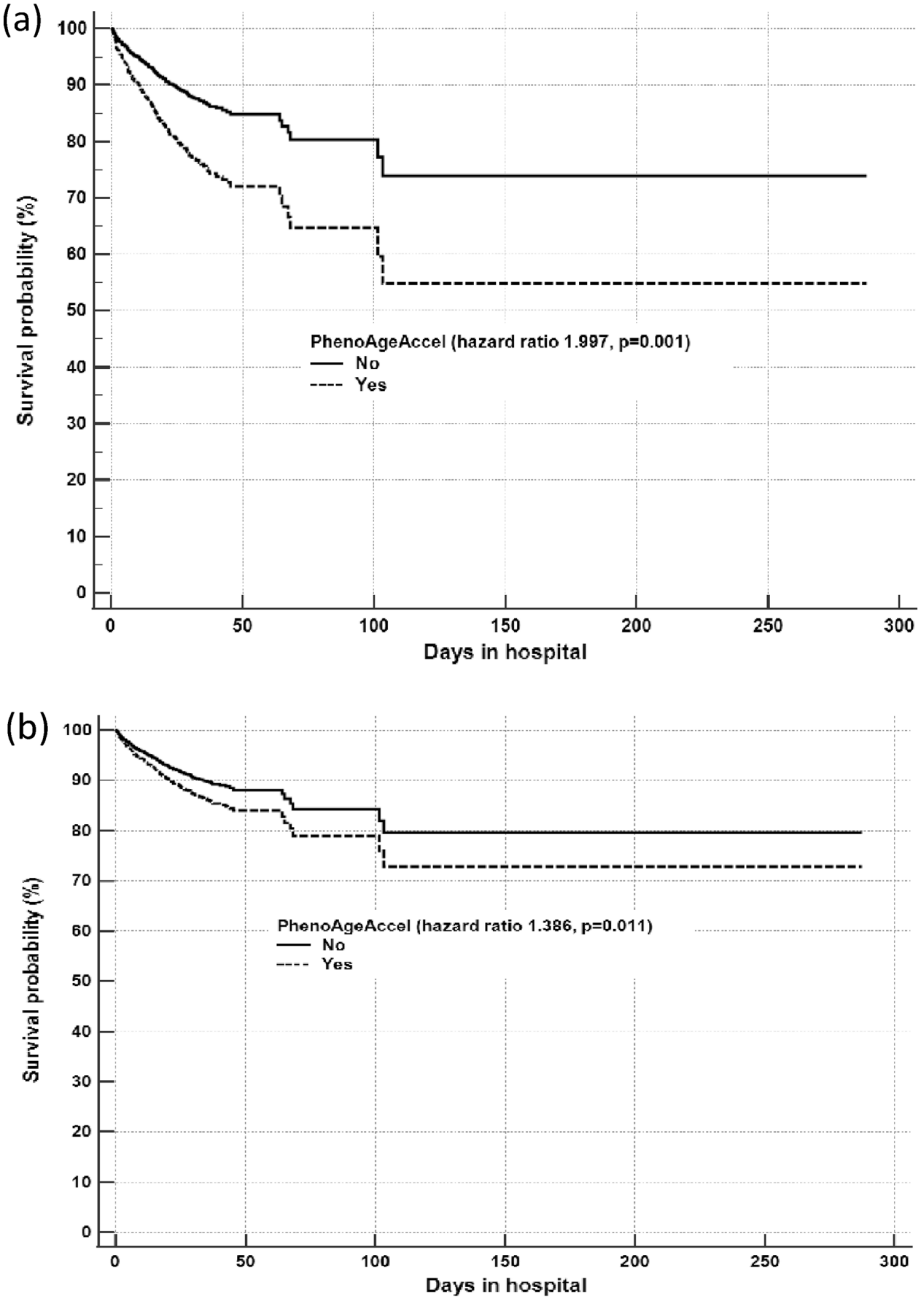
Difference in the survival curves between those with and without PhenoAgeAccel*. **a** Univariable analysis and **b** After adjusting for all the confounders including chronological age, elective surgery, chronic cardiovascular disease, chronic respiratory disease, end-stage renal failure, cirrhosis, immune disease, immunosuppressive therapy, metastatic cancer, lymphoma, leukemia, AIDS, and diabetes mellitus. *PhenoAgeAccel was present when the PhenoAge was older than chronological age after indexing one’s PhenoAge with the PhenoAge of the whole cohort

In this study, only patients who had the full set of nine blood tests done within 24 h of ICU admission were included. When any of these nine tests were repeated within the first 24 h of ICU admission, the one closest to the ICU admission time was used for consistency purposes.

### Statistical analysis

Descriptive statistics were used to describe the characteristics of the cohort, and categorical and continuous data with skewed distributions were analyzed by Chi Square and Mann–Whitney tests, respectively. Area under the receiver-operating-characteristic curve (AUROC) was first used to assess the discriminative ability of chronological age, PhenoAge and its related measures.

The association between PhenoAgeAccel and hospital mortality was then assessed by a stepwise forward Cox proportional hazards regression to examine the confounding effect of pre-existing chronic health conditions and severity of acute illness (using the Acute Physiology and Chronic Health Evaluation II score). Finally, we used a 4-knot restricted cubic spline function to assess whether residuals of phenotypical age more than chronological age could have a non-linear or plateauing effect on survival time of our patients [11].

Sample size calculation showed that 814 patients would give us 80% statistical power to confirm phenotypical age as a continuous predictor of mortality with an AUROC > 0.6, assuming the overall mortality rate of the cohort was 9% and a two-tailed p-value < 0.05 was taken as significant. To detect an AUROC > 0.65, only 363 patients would be needed. All data were analyzed by SPSS for Windows (version 24. IBM, USA), S-Plus (version 8.2, 2010, Insightful Corp., Seattle, Washington, USA), and MedCalc (version 20.218, 2023).

## Results

Of the 17,917 patients admitted to the ICU during the study period, 3175 admissions (17.7%) had the full set of blood tests to calculate patients’ ICU admission PhenoAge (Online supplement Fig. 2). These latter patients were slightly sicker compared to those without the full set of nine blood tests, with a lower proportion of patients who had undergone elective surgery (27.4% vs. 37.5%) with a slightly higher APACHE II score (15.0 vs 14.9) and hospital mortality (9.9% vs. 8.7%).

**Fig. 2:**
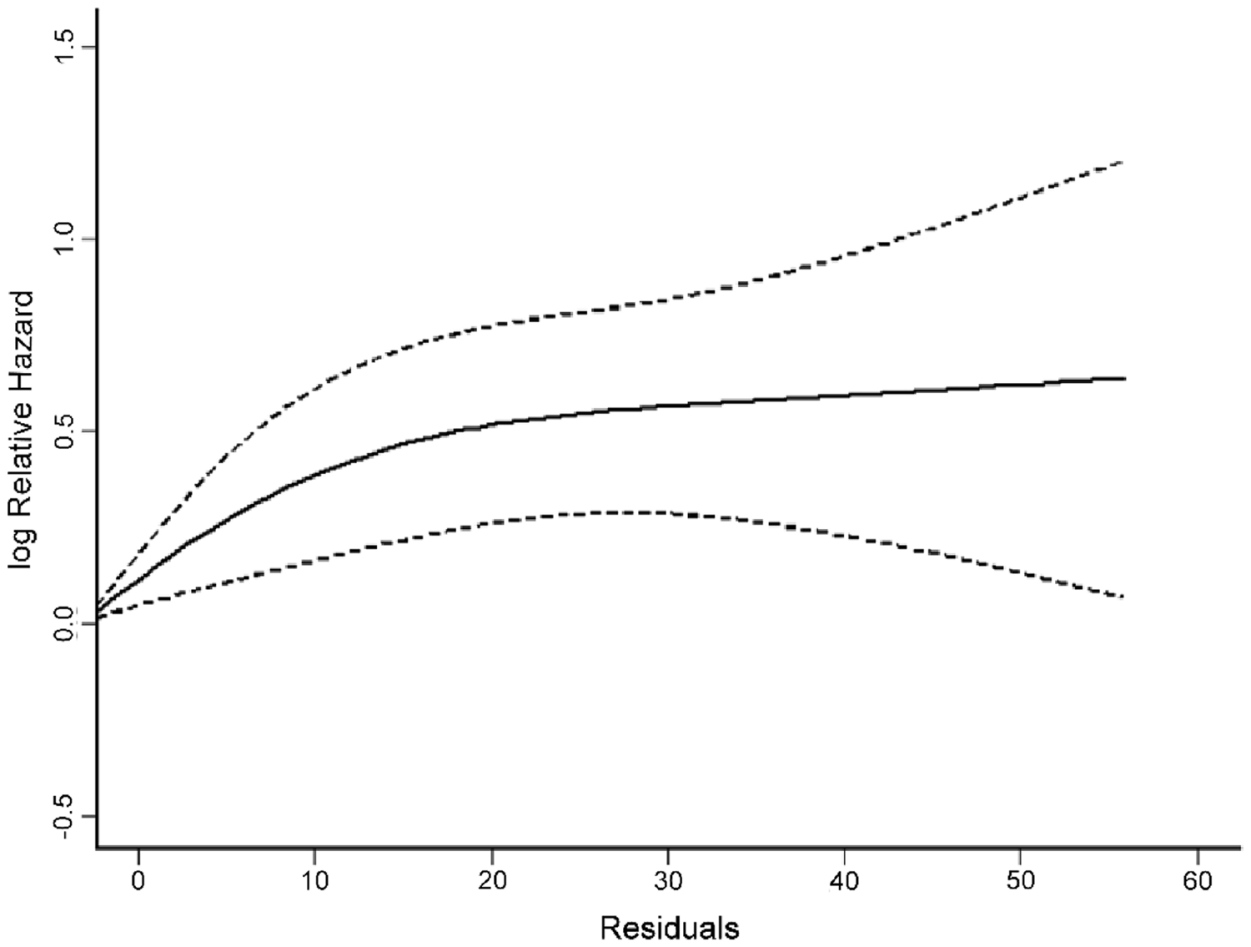
Non-linear association between residuals (in years) of regressing PhenoAge on chronological age and hazard of hospital mortality using a 4-knot restricted cubic spline function in the Cox proportional hazards regression. Dotted lines indicate 95% confidence interval

After excluding ICU readmissions during the same hospitalization (n = 225), 2950 patients were further analyzed in this study. As expected, emergency admissions, chronic respiratory and cardiovascular diseases, cirrhosis, immunosuppressive therapy, lymphoma, AIDS, and a high APACHE II score (of > 15) were more common among those who died after admission (Table 1). Chronological age was also significantly different between survivors and non-survivors (p = 0.008), so were PhenoAge and its related measures. However, the ability of the PhenoAge (AUROC 0.648) and its related measures were all statistically better than chronological age (AUROC 0.547) in differentiating between survivors and non-survivors (Online supplement Fig. 3).

Stepwise forward Cox regression showed that PhenoAgeAccel was significantly associated with an increased risk of mortality (hazard ratio [HR] 1.997, 95% confidence interval [CI] 1.568–2.542; p = 0.001) (Table 2) (Fig. 1a); and this adverse association remained significant (adjusted HR 1.386, 95% CI 1.077–1.784; p = 0.011) (Fig. 1b) after adjusting for multiple pre-existing medical conditions and severity of acute illness. The hazard associated with excessive phenotypical age compared to chronological age was ‘dose-related’ and this effect did not reach a plateau until one’s biological age was close to 20 years older than their chronological age (Fig. 2).

**Table 2:** Stepwise forward Cox proportional hazards regression to assess the association between PhenoAgeAccel* and hospital mortality while adjusting for important confounders

Predictor(s) in the model	Hazard ratio** (95%CI)	p value
PhenoAgeAccel*	1.997 (1.568–2.542)	0.001
PhenoAgeAccel + chronological age	1.982 (1.557–2.524)	0.001
PhenoAgeAccel + chronological age + elective surgery	1.672 (1.310–2.133)	0.001
PhenoAgeAccel + chronological age + elective surgery + chronic respiratory disease	1.676 (1.313–2.138)	0.001
PhenoAgeAccel + chronological age + elective surgery + chronic respiratory disease + chronic cardiovascular disease	1.650 (1.292–2.108)	0.001
PhenoAgeAccel + chronological age + elective surgery + chronic respiratory disease + chronic cardiovascular disease + cirrhosis	1.623 (1.270–2.074)	0.001
PhenoAgeAccel + chronological age + elective surgery + chronic respiratory disease + chronic cardiovascular disease + cirrhosis + end-stage renal failure	1.676 (1.308–2.147)	0.001
PhenoAgeAccel + chronological age + elective surgery + chronic respiratory disease + chronic cardiovascular disease + cirrhosis + end-stage renal failure + immune disease	1.678 (1.310–2.150)	0.001
PhenoAgeAccel + chronological age + elective surgery + chronic respiratory disease + chronic cardiovascular disease + cirrhosis + end-stage renal failure + immune disease + immunosuppressed	1.668 (1.302–2.137)	0.001
PhenoAgeAccel + chronological age + elective surgery + chronic respiratory disease + chronic cardiovascular disease + cirrhosis + end-stage renal failure + immune disease + immunosuppressed + AIDS	1.671 (1.304–2.141)	0.001
PhenoAgeAccel + chronological age + elective surgery + chronic respiratory disease + chronic cardiovascular disease + cirrhosis + end-stage renal failure + immune disease + immunosuppressed + AIDS + lymphoma	1.673 (1.306–2.144)	0.001
PhenoAgeAccel + chronological age + elective surgery + chronic respiratory disease + chronic cardiovascular disease + cirrhosis + end-stage renal failure + immune disease + immunosuppressed + AIDS + lymphoma + metastatic cancer	1.673 (1.306–2.144)	0.001
PhenoAgeAccel + chronological age + elective surgery + chronic respiratory disease + chronic cardiovascular disease + cirrhosis + end-stage renal failure + immune disease + immunosuppressed + AIDS + lymphoma + metastatic cancer + leukemia	1.672 (1.304–2.142)	0.001
PhenoAgeAccel + chronological age + elective surgery + chronic respiratory disease + chronic cardiovascular disease + cirrhosis + end-stage renal failure + immune disease + immunosuppressed + AIDS + lymphoma + metastatic cancer + leukemia + diabetes mellitus	1.720 (1.340–2.207)	0.001
Final full model ^#^		
PhenoAgeAccel	1.386 (1.077–1.784)	0.011
Chronological age	1.006 (0.998–1.013)	0.152
Elective surgery	0.421 (0.271–0.653)	0.001
Chronic respiratory disease	1.401 (0.902–2.176)	0.133
Chronic cardiovascular disease	1.163 (0.810–1.670)	0.414
Cirrhosis	1.474 (0.851–2.553)	0.166
End-stage renal failure	0.574 (0.332–0.992)	0.048
Immune disease	0.639 (0.308–1.327)	0.230
Immunosuppressed	1.053 (0.724–1.533)	0.786
AIDS	2.014 (0.475–8.544)	0.342
Lymphoma	1.192 (0.650–2.184)	0.571
Metastatic cancer	0.686 (0.362–1.671)	0.247
Leukemia	0.797 (0.380–1.671)	0.548
Diabetes mellitus	1.592 (1.016–2.494)	0.040
APACHE II score > 15	3.881 (2.703–5.574)	0.001

*PhenoAgeAccel is a dichotomized estimate to reflect whether the biological age of the patient is older than the chronological age while accounting for the PhenoAge of the whole cohort by regressing PhenoAge on chronological age; a positive residual means a patient’s phenotypical or biological age is older than their chronological age compared to other patients included in this study. CI, confidence interval. All comorbidities except diabetes mellitus are defined according to the Acute Physiology and Chronic Health Evaluation (APACHE) II model. ^#^The Harrell’s C index of the final full model was 0.746 (95%CI 0.719–0.773). **Hazard ratio was referring to mortality risk of the PhenoAgeAccel during the stepwise Cox regression, and each predictor in the final full model

Finally, analysis of the association between PhenoAgeAccel and comorbidities showed that PhenoAgeAccel was indeed more prevalent among those with severe pre-existing chronic cardiovascular disease, end-stage renal failure, cirrhosis, immune disease, diabetes mellitus, or those treated with immunosuppressive therapy (Table 3).

**Table 3:** Chronic health conditions in association with the presence of PhenoAgeAccel*

Chronic health conditions	Odds ratio (95%CI)	p value
Cardiovascular disease	1.899 (1.445–2.497)	0.001
Respiratory disease	1.284 (0.891–1.850)	0.180
End-stage renal failure	28.9 (12.686–65.838)	0.001
Cirrhosis	2.749 (1.623–4.656)	0.001
Immune disease	1.807 (1.103–2.960)	0.019
Immunosuppressed	1.826 (1.397–2.388)	0.001
AIDS	0.876 (0.146–5.248)	0.884
Lymphoma	1.104 (0.652–1.869)	0.713
Metastatic cancer	0.827 (0.598–1.145)	0.253
Leukemia	1.482 (0.860–2.554)	0.157
Diabetes mellitus	3.128 (2.316–4.224)	0.001

All comorbidities except diabetes mellitus are defined according to the Acute Physiology and Chronic Health Evaluation (APACHE) II model. *PhenoAgeAccel is a dichotomized estimate to reflect whether the biological age of the patient is older than the chronological age while accounting for the PhenoAge of the whole cohort by regressing PhenoAge on chronological age; a positive residual means a patient’s phenotypical or biological age is older than their chronological age compared to other patients included in this study. CI, confidence interval

## Discussion

This study showed that being phenotypically older than one’s chronological age was common among those with severe pre-existing medical conditions, and this was associated with an increased risk of mortality that was not fully captured or explained by comorbidities and severity of acute illness. These results have some clinical and research implications and require further discussion.

First, clinicians have long held an intuition that biological or phenotypical age is more important than chronological age in determining health outcomes, and chronological age is an imperfect marker of one’s physiological reserve or ‘effective age’ [10, 12]. With intensive computing capability including the use of artificial intelligence, a few phenotypical or biological age estimation models that are predictive of long-term functional and survival outcomes in a wide range of clinical settings have been developed [7–10, 13–16]. The utility of biological age in acute care setting has, however, not been thoroughly assessed. Our results confirmed the clinicians’ intuition that phenotypical age was more predictive of mortality than chronological age, even though on its own PhenoAge was still far less predictive of mortality compared to the APACHE II model. Nonetheless, the adverse effect of being biological older than expected did appear to add predictive value to severity of acute illness and comorbidities in predicting mortality. The reasons why PhenoAgeAccel is potentially useful in addition to the APACHE II model might be because PhenoAgeAccel is indexed to the rest of patients included in the study within the same context, such that any significant changes in the blood chemistry used to assess PhenoAgeAccel were calibrated in reference to other critically ill patients in the same critical care setting, making it less likely to be affected by acute inflammatory response and acute resuscitation such as fluid therapy. For this reason, PhenoAgeAccel did not exist in our patients until their PhenoAge was at least 33.4 years older than their corresponding chronological age (Online supplement Figure 1a). Furthermore, diabetes mellitus [17], C-reactive protein [18], lymphocyte count, red blood cell width distribution [19, 20], as well as mean red blood cell volume [21], are not included as predictors in the APACHE II model. If our results can be confirmed by other centers, use of phenotypical age instead of chronological age as a risk adjustment tool for clinical audits and research on critically ill patients should be seriously considered.

Second, our results showed that the difference between PhenoAge and chronological age after indexed to the local context had a relatively linear ‘dose-related’ relationship to hospital mortality that did not reach a plateau until up to a 20-year gap. Emerging studies have demonstrated that phenotypical age is more like a dynamic clock [5]; this would also mean that phenotypical age is like a quantitative assessment of physiological reserve, and there is an opportunity for interventions to ‘dial back’ the phenotypical age of critically ill patients, like non-critically ill patients [22, 23]. A recent study also showed that Clonal Hematopoiesis of Indeterminate Potential (CHIP) had a direct correlation with biological age, including the PhenoAge [3], providing strong biological rationale to support the prognostic significance of PhenoAge. We argue that it is scientifically valid to use phenotypical age as one of the secondary outcomes when assessing effectiveness of medical interventions in phase II clinical trials involving critically ill patients.

Third, we showed that PhenoAgeAccel was associated with a number of comorbidities. This result was not surprising because PhenoAge estimation requires some blood parameters (e.g. blood glucose and alkaline phosphatase) that are also often abnormal in some comorbidities (such as diabetes mellitus and cirrhosis, respectively). Medical advances have prolonged lifespan of many individuals who have serious chronic illnesses including HIV infection [24]. Our next clinical challenge is to improve health span by reducing biological age, and reduce frailty and the burden of chronic health conditions for the aging population [1, 6, 25].

Finally, we need to acknowledge the limitations of this study. Although the sample size of this study was not small, it is a single-center observational study and selection bias is probable. The selected patients represented only a small proportion of all admissions (17.7%) and they were slightly sicker than those who did not have the full set of nine blood tests to allow us to estimate their PhenoAge. Confirmation of the external validity our results by a prospective multicenter study is, therefore, needed. As we alluded to earlier, serial measurements of phenotypical age will be a useful addition in any prospective studies because it would help us to understand how phenotypical age would change during critical illness (or a lack of it), and whether interventions, such as intensive nutritional support or physical rehabilitation, could improve it [6, 25]. Recent studies suggested that biological age is likely related to frailty in the elderly [26], and frailty could be even more important than markers of DNA methylation in predicting long-term health outcomes [4]. Whether biological age remains important as a prognostic factor after accounting for one’s frailty in the critically care setting remains unknown, and we plan to investigate this important issue in our next study. The Levine PhenoAge model belongs to the second-generation DNA methylation age algorithm [27]. A number of other biological age models including the third-generation DNA methylation age algorithms have been published since [13]; whether one model is superior to the other biological age models (e.g. GrimAge model) and also Clonal Hematopoiesis of Indeterminate Potential (CHIP) in predicting mortality of the critically ill has not been investigated [3, 28, 29], but this requires further study.

In conclusion, this proof of concept study showed that being phenotypically older than one’s chronological age after indexed to the local study context – also called PhenoAgeAccel – was common among those with severe comorbidities. The divergence between biological and chronological age had a relatively linear ‘dose-related’ relationship with the risk of hospital mortality in the critically ill that was not fully explained or captured by comorbidities and severity of acute illness. Further studies are needed to assess whether medical interventions could reduce biological age [25], ultimately improving patient-centered outcomes.

### Supplementary Information

Below is the link to the electronic supplementary material.

Supplementary file1 (DOCX 466 KB)

## Data Availability

It will be available for reasonable requests.
