# Supplementary material for: Biological age is superior to chronological age in predicting hospital mortality of the critically ill
Source: Intern Emerg Med. 2023 Aug 28;18(7):2019–28. doi: 10.1007/s11739-023-03397-3 (PMC10543822; doi:10.1007/s11739-023-03397-3)
Supplement: Supplementary file 1 — Supplementary file1 (DOCX 466 KB) [file 11739_2023_3397_MOESM1_ESM.docx]

**sFigure 1.** (a) Histogram showing the distribution of the residuals after regressing PhenoAge on chronological age and (b) the scatter plot showing the relationship between PhenoAge and chronological age. PhenoAgeAccel was present when PhenoAge was older than chronological age after indexing each patient’s PhenoAge with the PhenoAge of the whole study cohort.

(a)


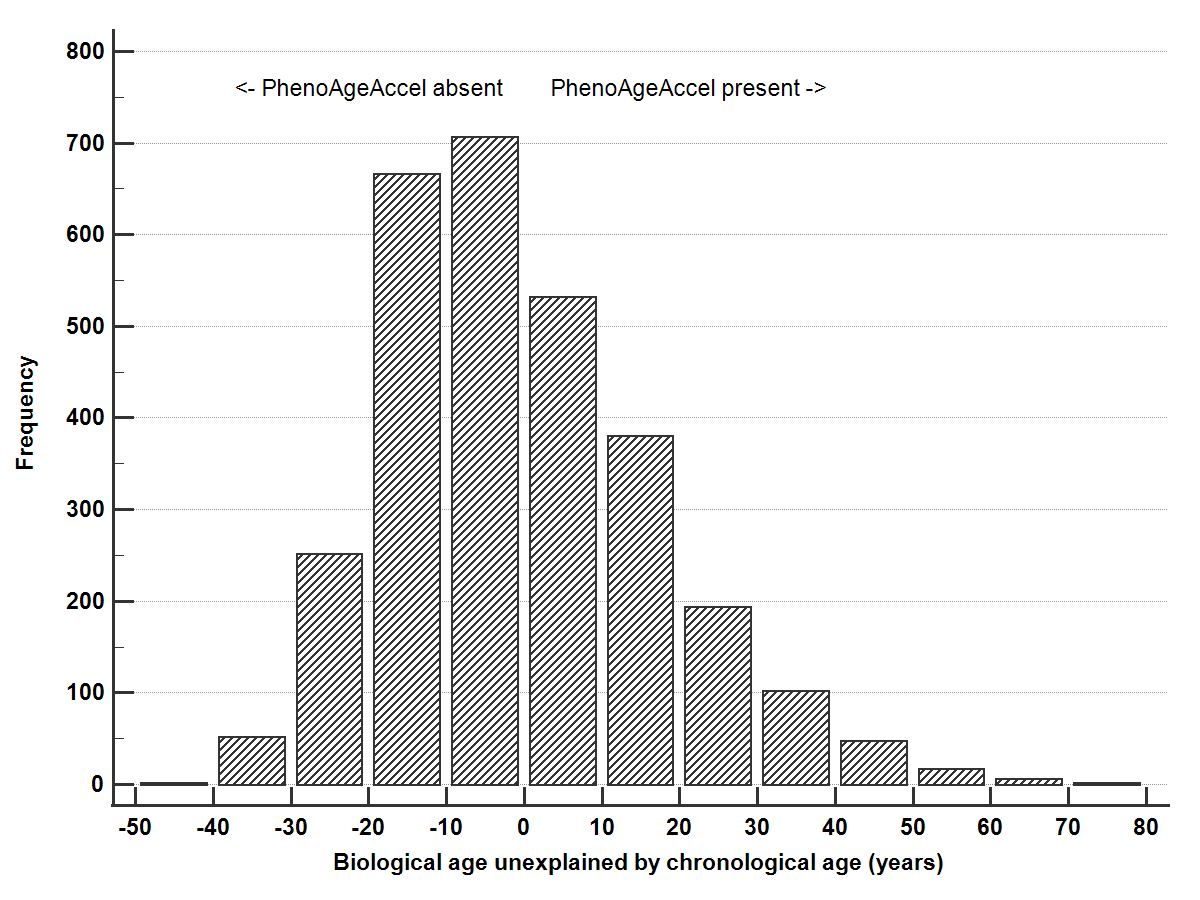


(b)
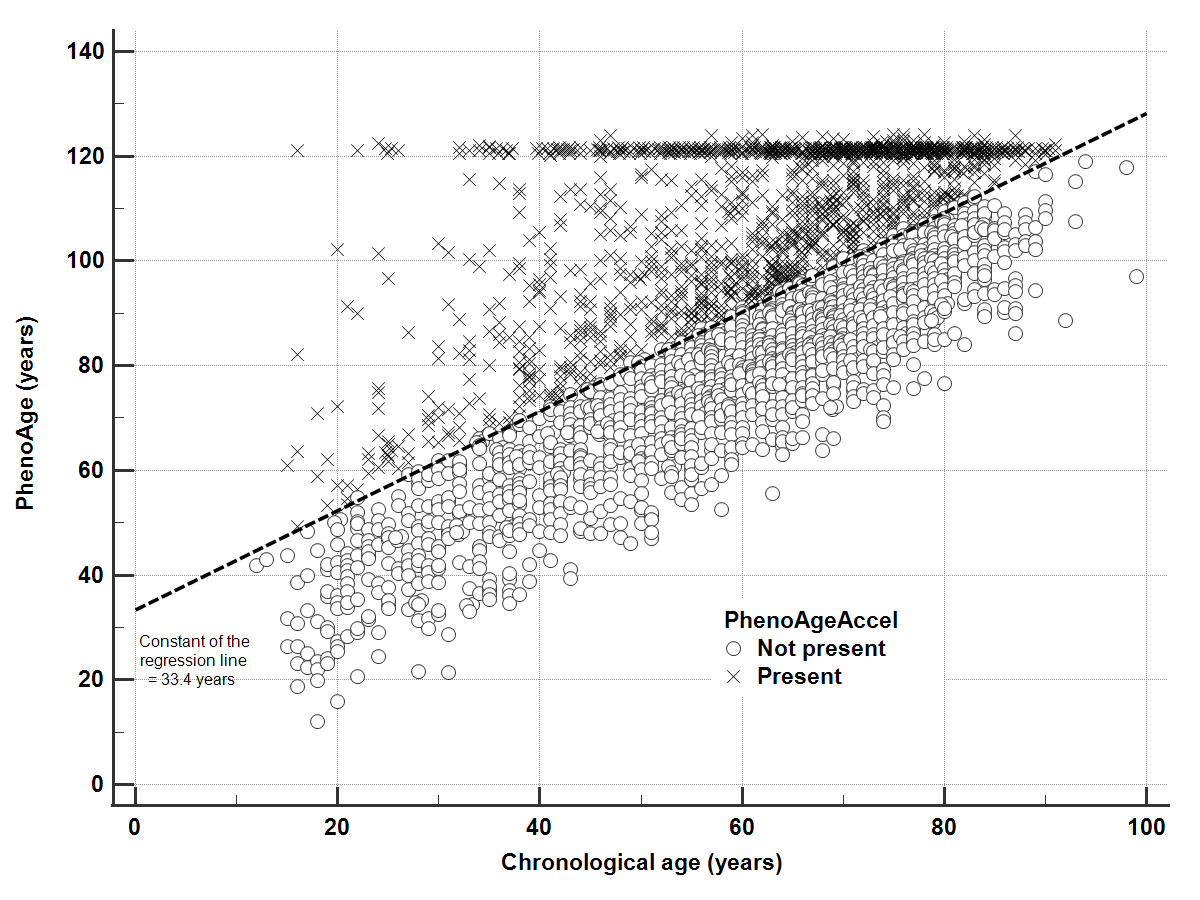


**sFigure 2.** Flow chart showing inclusion and exclusion of patients for the study.


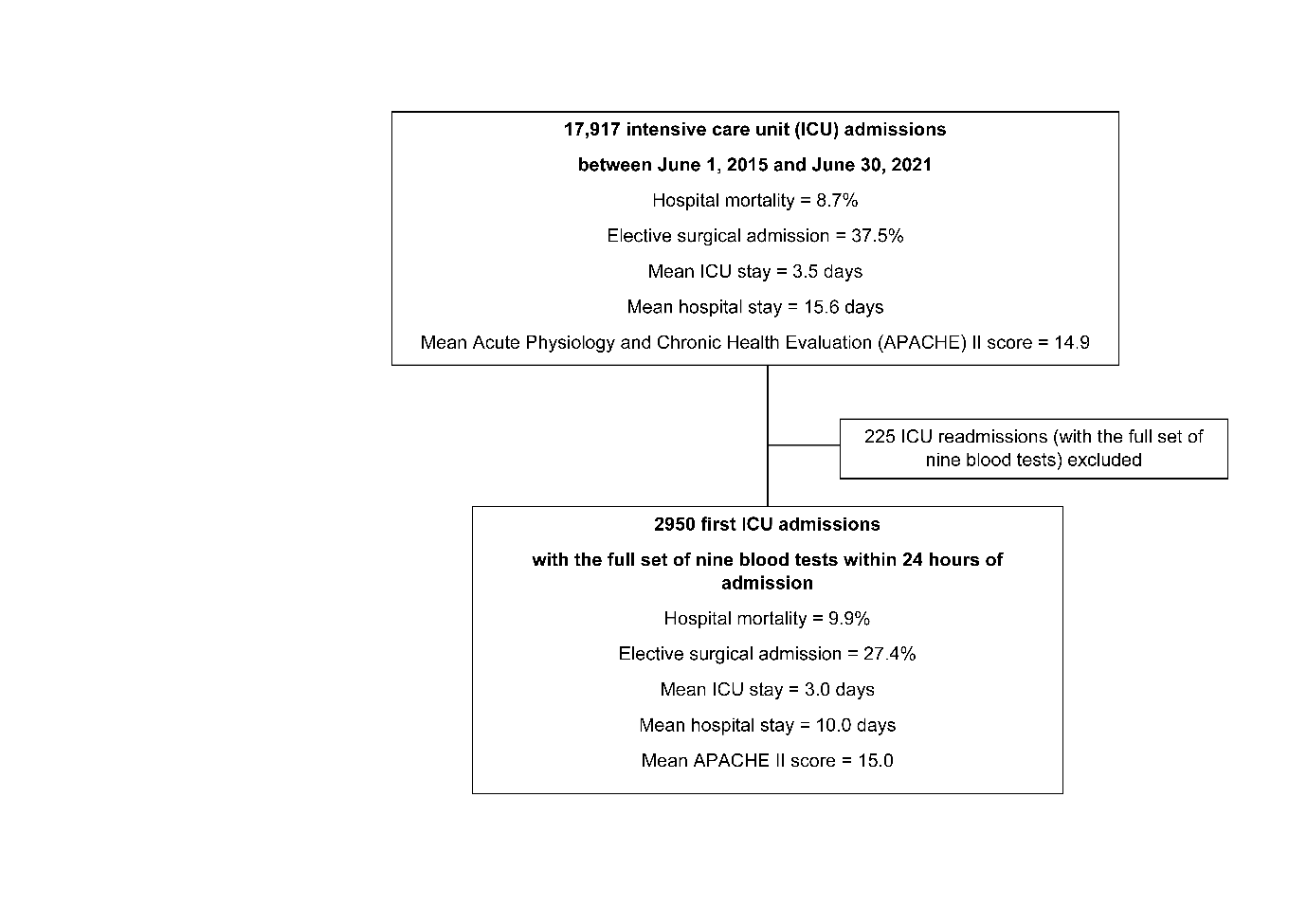


**sFigure 3.** The ability of PhenoAge, the residuals from regressing PhenoAge on chronological age, absolute difference between chronological age and PhenoAge, and chronological age to differentiate between hospital survivors (n=2659) and non-survivors (n=291).
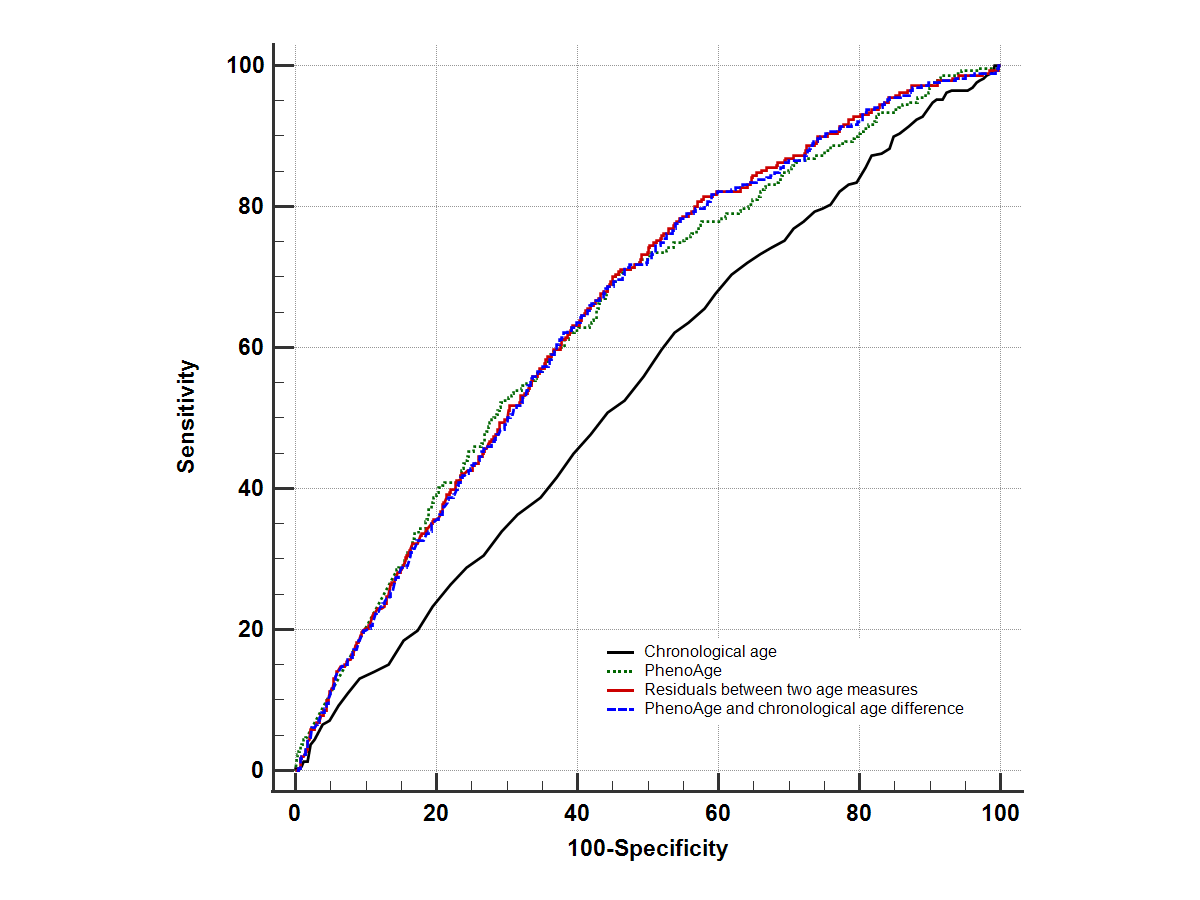


| **Variable** | **Area under the receiver operating characteristic curve (AUROC)** | **Standard Error** | **95% confidence interval** |
| --- | --- | --- | --- |
| Chronological age | 0.547 | 0.0174 | 0.513-0.581 |
| PhenoAge | 0.648 | 0.0167 | 0.615-0.681 |
| Absolute difference between PhenoAge and chronological age | 0.651 | 0.0162 | 0.620-0.683 |
| Residuals after regressing PhenoAge on chronological age | 0.654 | 0.0161 | 0.623-0.686 |

All PhenoAge related measures were significantly better than chronological age in differentiating between hospital survivors and non-survivors (with all p values <0.001).

**sTable 1.** Equations used to calculate the PhenoAge of the patients by using the nine blood test results (together with their chronological age) on admission to the intensive care unit.

| **Linear predictor =** | Albumin (g/L) x -0.0336 + creatinine (umol/L) x 0.0095 + glucose (mmol/L) x 0.1953 + Ln [C-reactive protein (mg/dL)] x 0.0954 + lymphocyte percentage (%) x -0.012 + mean red blood cell volume (fL) x 0.0268 + red blood cell distribution width (%) x 0.3306 + alkaline phosphatase (U/L) x 0.00188 + white blood cell count (10^3 cells/mL) x 0.0554 + chronological age (years) x 0.0804 + b_0_ coefficient |
| --- | --- |
| **b_0_ coefficient** **=** | -19.907 |
| **Mortality risk =** | 1-EXP(-EXP[linear predictor)*1.51714/0.0076927] |
| **PhenoAge =** | 141.50 + LN[-0.00553*LN(1 – Mortality risk)]/0.09165 |
| **References used to extract these equations:** | 1. Levine ME, Lu AT, Quach A, Chen BH, Assimes TL, Bandinelli S, Hou L, Baccarelli AA, Stewart JD, Li Y, Whitsel EA, Wilson JG, Reiner AP, Aviv A, Lohman K, Liu Y, Ferrucci L, Horvath S. An epigenetic biomarker of aging for lifespan and healthspan. *Aging (Albany NY)* 2018;10(4):573-591. doi: 10.18632/aging.101414. 2. Liu Z, Kuo PL, Horvath S, Crimmins E, Ferrucci L, Levine M. A new aging measure captures morbidity and mortality risk across diverse subpopulations from NHANES IV: A cohort study. *PLoS Med* 2018;15(12):e1002718. doi: 10.1371/journal.pmed.1002718. 3. Liu Z, Kuo PL, Horvath S, Crimmins E, Ferrucci L, Levine M. Correction: A new aging measure captures morbidity and mortality risk across diverse subpopulations from NHANES IV: A cohort study. *PLOS Med* 2019;16(2): e1002760. |
